# Other PHAC publications

**DOI:** 10.24095/hpcdp.45.9.07

**Published:** 2025-09

**Researchers from the Public Health Agency of Canada also contribute to work published in other journals and books. Look for the following articles published in 2025:**

Gagnon I, Turner M, Lacasse-Courchesne A, McKee M, Tang ML, Sajjadi M, **Friedman D**, et al. Feasibility of direct-access physical therapy for concussion management in the pediatric emergency department: a pilot implementation study. Phys Ther. 2025;105(5):pzaf051. https://doi.org/10.1093/ptj/pzaf051

Langevin P, Plotnick LH, Turner M, **Friedman D**, Agnihotram R, Greenstone I, et al. Direct-access physiotherapy to improve access to quality care for children and adolescents presenting to the pediatric emergency department with musculoskeletal problems: the PEDPT-MSK pilot randomized control trial. J Orthop Sports Phys Ther. 2025;55(6):386-450. https://doi.org/10.2519/jospt.2025.13321

Marcellus L, Jack SM, MacKinnon K, Hill ME, Gonzalez A, Campbell K, […] **Tonmyr L**, et al. Strategies to engage and retain pregnant individuals and young mothers in the nurse-family partnership program (Canada): an interpretive descriptive study. Child Abuse Negl. 2025;167:107537. https://doi.org/10.1016/j.chiabu.2025.107537

Nevill AM, **Lang JJ**, Niemz M, Tomkinson GR. How should youth handgrip strength be normalized? New insights using 3-D allometry with “generalizable” norm-referenced values, data from NHANES. Sports Med. 2025:101012. https://doi.org/10.1007/s40279-025-02235-0

**Prince SA, Thomas T**, Apparicio P, Rodrigue L, Jobson C, Walker KL, **Butler GP, Wasfi R**. Cycling infrastructure as a determinant of cycling for recreation and transportation in Montral, Canada: a natural experiment using the longitudinal national population health survey. Int J Behav Nutr Phys Act. 2025;22(1):71. https://doi.org/10.1186/s12966-025-01767-y

Raza SZ, Whitten C, **Randell S**, Sparkes B, Denic N. Polysubstance toxicity deaths in Newfoundland and Labrador: a retrospective study. J Public Health (Oxf.). 2025;47(2):114-22. https://doi.org/10.1093/pubmed/fdaf001

**Tanaka M, Pollock NJ, Shields M, Richter S, Blair D-L**, Cormier F, […] **Tonmyr L**. Referrals to child and family services during the COVID-19 pandemic: an analysis of administrative data from British Columbia and Northwest Territories, Canada. Child Abuse Negl. 2025;167:107517. https://doi.org/10.1016/j.chiabu.2025.107517

Zuniga YM, Zumla A, Zuhlke LJ, Zoladl M, Ziaeian B, Zhong C, […] **Lang JJ**, […] **Badawi A**, et al. Characterising acute and chronic care needs: insights from the Global Burden of Disease Study 2019. Nat Commun. 2025;16(1):4235. https://doi,org/10.1038/s41467-025-56910-x

